# Activation of Indoleamine 2,3-Dioxygenase in Patients with Scrub Typhus and Its Role in Growth Restriction of *Orientia tsutsugamushi*

**DOI:** 10.1371/journal.pntd.0001731

**Published:** 2012-07-31

**Authors:** Thanavadee Prachason, Kanittha Konhan, Piyapat Pongnarin, Somruedee Chatsiricharoenkul, Yupin Suputtamongkol, Chanin Limwongse

**Affiliations:** 1 Division of Molecular Genetics, Department of Research and Development, Faculty of Medicine, Siriraj Hospital, Mahidol University, Bangkok, Thailand; 2 Department of Immunology, Faculty of Medicine, Siriraj Hospital, Mahidol University, Bangkok, Thailand; 3 Department of Pharmacology, Faculty of Medicine, Siriraj Hospital, Mahidol University, Bangkok, Thailand; 4 Division of Infectious Diseases, Department of Internal Medicine, Faculty of Medicine, Siriraj Hospital, Mahidol University, Bangkok, Thailand; 5 Division of Medical Genetics, Department of Internal Medicine, Faculty of Medicine, Siriraj Hospital, Mahidol University, Bangkok, Thailand; Aix-Marseille Université, Faculté de Médecine, France

## Abstract

**Background:**

Our earlier genome-wide expression study revealed up-regulation of a tryptophan-catabolizing enzyme, indoleamine 2,3-dioxygenase (IDO1), in patients with scrub typhus. This gene has been previously reported to have anti-microbial activity in a variety of infectious diseases; therefore, we aimed to prove whether it is also involved in host defense against *Orientia tsutsugamushi* (OT) infection.

**Methodology/Principal Findings:**

Using LC-MS, we observed an increased ratio of serum L-kynurenine to serum L-tryptophan in patients with scrub typhus, which suggests an active catalytic function of this enzyme upon the illness. To evaluate the effect of IDO1 activation on OT infection, a human macrophage-like cell line THP-1 was used as a study model. Although transcription of IDO1 was induced by OT infection, its functional activity was not significantly enhanced unless the cells were pretreated with IFN-γ, a potent inducer of IDO1. When the degree of infection was evaluated by quantitative real-time PCR, the relative number of OT 47 kDa gene per host genes, or infection index, was markedly reduced by IFN-γ treatment as compared to the untreated cultures at five days post-infection. Inhibition of IDO1 activity in IFN-γ treated cultures by 1-methyl-L-tryptophan, a competitive inhibitor of IDO1, resulted in partial restoration of infection index; while excessive supplementation of L-tryptophan in IFN-γ treated cultures raised the index to an even higher level than that of the untreated ones. Altogether, these data implied that IDO1 was partly involved in restriction of OT growth caused by IFN-γ through deprivation of tryptophan.

**Conclusions/Significance:**

Activation of IDO1 appeared to be a defensive mechanism downstream of IFN-γ that limited intracellular expansion of OT via tryptophan depletion. Our work provided not only the first link of in vivo activation of IDO1 and IFN-γ-mediated protection against OT infection but also highlighted the promise of this multifaceted gene in scrub typhus research.

## Author Summary

Scrub typhus is a potentially life-threatening infectious disease that is a major cause of acute undifferentiated fever in Asia-Pacific region. It is caused by *Orientia tsutsugamushi* (OT), an obligatory intracellular gram-negative bacterium in family rickettsiaceae. Earlier studies have shown that IFN-γ is essential in protection against OT infection in animals and cell-based models, but molecular mechanisms underlying such phenomenon remain largely unclear. In the present study, we are the first to demonstrate that activation of IDO1, a key enzyme in tryptophan-degrading pathway, is a mechanism downstream to IFN-γ in control of OT infection. IDO1 was found active in patients with acute scrub typhus. Subsequent in vitro experiments suggested that IDO1 restrains OT growth via deprivation of tryptophan, an important material for proliferation of OT, rather than via production of its downstream anti-microbial metabolites. Since IDO1 is a multifaceted gene that also plays a part in several processes of the immune system, including induction of tolerogenic responses; other possible consequences of IDO1 activation deserve further investigation in a more complex experimental model as well as in naturally infected human before subsequent clinical implication of this basic knowledge can be ultimately applied.

## Introduction

Scrub typhus is a potentially life-threatening infectious disease caused by *Orientia tsutsugamushi* (OT), an obligate intracellular gram-negative bacterium transmitted to human through the bite of a larval trombiculid mite. The disease is one of major public health problems in Asia-Pacific region, where approximately one billion people are at risk, and one million cases are affected each year [1]. Exposure to the field where its host vectors inhabit is an important risk factor for the infection especially among people who live in rural areas or work in a farm. So far, no effective strategy has ever succeeded in providing long lasting immunity to this particular infection [2].

Earlier studies have shown that IFN-γ is essential in protection against OT infection in animal and cell-based models [3], [4], [5]. Apart from its well-known roles in macrophage activation and the development of type I immune response, it was found to directly inhibit intracellular growth of OT in non-immune host cells [6]. Nevertheless, the underlying molecular mechanisms remain unclear. In natural infection of scrub typhus, a marked elevation of IFN-γ was consistently observed in acute serum of the infected patients [7], [8], [9]. Accordingly, our recently published genome-wide expression data in patients with scrub typhus have revealed that IFN-γ and a number of IFN-related genes are up-regulated during acute phase of the illness [10]. Among these, *indoleamine 2,3-dioxygenase-1* (*IDO1*) is an interesting gene with a growing number of studies for its multifaceted roles in the immune system. It encodes an intracellular heme-containing dioxygenase [11] that is an initial and rate-limiting enzyme of kynurenine pathway, a major catabolic pathway of tryptophan. Since tryptophan is the rarest essential intracellular amino acid; activation of IDO1, which leads to deprivation of tryptophan, becomes a potential strategy of the host to control the population of an invading microorganism. This notion was first proven in various cell models of toxoplasma infection, including human glioblastoma cell line [12], primary endothelial cells [13], uroepithelial cells [14], as well as monocyte-derived macrophages [15]. Later on, IDO1-mediated microbial-stasis was also reported in a number of intracellular and extracellular infections; including those caused by *Chlamydia spp.* [16], *Mycobacterium avium* [17], herpes simplex virus [18], [19], measles virus [20], cytomegalovirus [21], dengue virus [22], group B streptococcus [23], enterococci [24], and *Staphylococcus aureus* [25].

In addition, IDO1 also exerts an indirect antimicrobial activity via production of some certain downstream catabolites of kynurenine pathway [26], [27], [28]. For example, 3-hydroxy-kynurenine, a subsequent product of IDO1 activation, was found to limit replication of *Trypanosoma cruzi* in vivo; treatment of the infected mice with the metabolite can improve resistance to the infection as well as survival of the host [26]. Other downstream catabolites, including picolinic acid, 3-hydroxyanthranilic acid, and quinolinic acid, can also inhibit the growth of methicillin-resistant *S. aureus*, *S. epidermidis*, *Escherichia coli*, and multidrug-resistant *Pseudomonas aeruginosa* in vascular allograft [27].

Despite its protective role in a variety of infections, IDO1 activation paradoxically appears to be involved in suppression of the immune responses. This was first revealed by a key study showing that IDO1-mediated tryptophan degradation prevents allogeneic fetal rejection in mice [29]. It was later demonstrated in vitro that the enzyme exerts anti-proliferative effects on T cells, NK cells, as well as tumor cells via degradation of tryptophan and production of downstream metabolites, resembling its impact on microorganisms [30], [31], [32], [33]. Indeed, IDO1 expression in certain dendritic cell subsets was found to induce tolerogenic responses to antigenic stimuli through a variety of mechanisms; including induction of T cell anergy [34], apoptosis [35] and regulatory T cell differentiation [36]. When such concept is applied to a scenario of infection, it seems that IDO1 activation in some certain settings may contribute to ineffective development of adaptive immunity and allow a microorganism to persist. For example, HIV-induced IDO1 activation in peripheral blood of HIV-infected patients, which mainly contributed by plasmacytoid dendritic cell subpopulation, appeared to be responsible for unresponsiveness of CD4-positive T cell to stimulation of T cell receptor in vitro [37]. Moreover, levels of IDO1 expression in peripheral blood mononuclear cells from these patients also correlates with their viral loads; which further supports a link between IDO1, T cell dysfunction, and ineffective viral control [37].

In rickettsial infection, it was reported that IFN-γ-mediated IDO1 activation inhibited the growth of *Rickettsia conorii* in vitro, and such restriction could be relieved by tryptophan supplementation [38]. In contrast, proliferation of *Rickettsia prowazekii* was insensitive to IDO1-mediated tryptophan depletion [39]. In vivo up-regulation of IDO1, which positively correlated with IFN-γ and TNF-α expression, was observed at the skin lesion of patients with Mediterranean spotted fever, an illness caused by *R. conorii* infection [40]. However, the role of IDO1 in scrub typhus has never been directly investigated.

We hypothesized that up-regulation of IDO1 upon infection with scrub typhus could lead to rescriction of OT growth in host cells according to the fact that OT lacks an enzyme to generate tryptophan, which is an essential material for its expansion [41]. Since monocytes are the main source of active IDO1 in human peripheral blood [42], [43] and also seem to be a potential target of infection to carry the intracellular organisms to remote organs, a human macrophage cell line THP-1 was used as a model in subsequent in vitro infection experiments.

In the present study, we reported that IDO1 acitivity was increased in patients with scrub typhus. Activation of IDO1 by IFN- γ resulted in a lower number of OT load in THP-1 macrophages, and the degree of infection could be partly restored by an inhibitor of IDO1 enzyme. Supplementation with high-dose tryptophan did not only reverse the suppression of OT growth but markedly increased the number of OT in IDO1 active cultures. Our work not only provided the first link of in vivo activation of IDO1 and IFN-γ-mediated protection against OT infection but also highlighted the promise of this multifaceted gene in scrub typhus research.

## Materials and Methods

### Ethics statement

The study was conducted after obtaining the approval of the Ethics Committee of Faculty of Medicine Siriraj Hospital, Mahidol University, Bangkok, Thailand. Informed and written consent was derived from all patients and control subjects before their blood samples were collected.

### Patients

Serum samples were derived from patients with acute undifferentiated fever at the first visit to Siriraj Hospital and were stored at −80°C until being used. As a control group, serum samples from ten healthy blood donors were derived and handled in a similar manner. As detected by an indirect immunofluorescent assay, definite diagnosis of scrub typhus was made according to the following criteria: 1) presence of OT-specific antibody at a titer of ≥1∶400 in a single acute sera; or 2) ≥four-fold rising of OT-specific antibody in paired sera collected two weeks apart [44]. Only confirmed cases of scrub typhus were selected to undergo LC-MS analysis, and their clinical and laboratory data were retrospectively reviewed.

### LC-MS analysis of L-tryptophan (L-Trp) and L-kynurenine (L-Kyn)

Analysis of L-Trp and L-Kyn was performed using a validated high performance liquid chromatography with tandem mass spectrometry (HPLC-MS/MS) method in accordance with the USFDA guidelines [U.S. Department of Health and Human Services Food and Drug Administration Center for Drug Evaluation and Research 2001]. Briefly, 3-nitro-L-tyrosine was added to each sample as an internal standard, and protein precipitation was performed using 10% trichloroacetic acid. Chromatographic separation of subsequent organic layer was carried out on LC-MS/MS with C18, 2.5 µm (50×3.00 mm i.d.). A mobile phase consisting of acetonitrile and 0.1% formic acid (Gradient condition) was delivered at a flow rate of 0.2 ml/min. Mass spectra were obtained using a Quattro Premier XE mass spectrometer (Micromass. UK), operated in multiple reaction monitoring mode. Sample introduction and ionization was performed by electrospray ionization in the positive ion. The mass transition ion-pair for L-Trp [*M*+H]+ and L-Kyn L-Trp [*M*+H]+ ions was selected as *m*/*z* 205.08>188.00 and 205.08>146.08 respectively. The mass transition ion-pair for 3-nitro-L-tyrosine [*M*+H]+ ions was selected as *m*/*z* 227.02>181.03. The data acquisition was ascertained by Masslynx 4.1 software. The detectable ranges of L-Trp and L-Kyn in both human serum and culture media were 0.05–50 µg/ml and 0.15–10 µg/ml respectively. The best linear fit was achieved with a 1/x weighting factor, showing a mean correlation coefficient (*r* ^2^)≥0.998.

### Preparation of OT stock

OT Standard Kato strain (CSUR R163) was propagated using mouse fibroblast cell line L929 as a host. When more than 90% of the host cells were infected, as determined by Giemsa staining, the media was replaced with fresh media. The infected cells were then dislodged from the flask and broken down using 1.0-mm-diameter glass beads with vigorous vortexing. The disrupted cell suspension ensuing from multiple parallel cultures were pooled together and stored as multiple small aliquots in liquid nitrogen. Before being used, a few aliquots of frozen OT inoculum were thawed in water bath at 37°C. Afterwards, the cell suspension was thoroughly mixed and broken down once again with glass beads and vortex before being centrifuged at 500×g for five minutes to sediment host cell lysate. Subsequent OT-containing supernatant was immediately used to infect THP-1 as well as to determine the infectivity of the inoculum by infected cell counting method with some modifications [45], [46]. Briefly, two-fold serial dilution of OT-containing inoculum was inoculated onto L929 monolayers on the glass coverslips. After 24 hours of incubation, the degree of infection was determined using an indirect immunofluorescence assay. Infected-cell-counting unit (ICU) was calculated according to the following formula: ICU = (total number of cells used in infection)×(ratio of infected cells to counted cells)×(dilution fold of OT inoculum) [45].

### Cell culture and in vitro infection

Twenty-four hours prior to an in vitro infection experiment, 2×10^5^ cells of THP-1 cell suspension were transferred into each well of 24-well plate and treated with 100 nM pharbol 12-myristate 13-acetate (PMA) (Sigma-Aldrich) to induce cell adherence. When IDO1 expression was required, the cells were be treated with 20 ng/ml IFN-γ (R&D Systems, Minneapolis, MN), along with 1 mM 1-methy-L-tryptophan (1-MT) (Sigma-Aldrich), L-Trp (Sigma-Aldrich) supplement, or neither. On the day of the experiments, the culture media was aspirated; and OT-containing inoculum of 5.9×10^5^ ICU, prepared as described in the previous section, was inoculated onto the THP-1 monolayer. Infection process was facilitated by centrifugation at 1,450×g for five minutes and further incubation in a humidified 5% CO_2_ atmosphere at 37°C for one hour. The inoculum was then replaced with fresh PMA-containing media, together with additional treatments corresponding to each pre-infectious condition; this time point was designated as zero hour post-infection (p.i.). At specified time points, culture media was collected for determination of IDO1 activity or just discarded. The cell layer was then rinsed three times with phosphate buffer saline to wash out extracellular organisms. Finally, the infected cells were collected and further processed for RNA or DNA study.

### Assessment of IDO1 expression by quantitative real-time PCR (qPCR)

Total RNA was extracted from each cell culture that had been lyzed in Trizol reagent (Invitrogen, Calsbad, CA) in accordance to the manufacturer's instruction. cDNA was synthesized from subsequent RNA extract using SuperScript III First-Strand Synthesis System (Invitrogen, Carldbad, CA). Specific amplification of TATA binding protein (TBP) and IDO1 gene transcripts was performed in duplicate using LightCycler FastStart DNA Master SYBR Green I reagents (Roche Applied Science), two µL of an appropriate dilution of a cDNA sample, and a specific primer pair for *TBP* or *IDO1*. The primer sequences were given in Table S1. Relative quantification analysis of qPCR data was then performed by LightCycler 480 software (Roche Applied Science) based on comparative ΔΔCt method. *IDO1* and *TBP* were set as the target gene and the reference gene respectively. Finally, a mean expression ratio of *IDO1* and *TBP* in each culture was normalized to that of mock-infected culture harvested at 0 hour p.i..

### Assessment of OT growth by qPCR

DNA was isolated from infected cell cultures using standard phenol/chloroform method [47]. Specific amplification of human methylenetetrahydrofolate reductase (MTHFR) gene and OT 47 kDa gene was performed in triplicate using LightCycler FastStart DNA Master SYBR Green I reagents (Roche Applied Science), two µL of an extracted DNA sample, and a specific primer pair for *MTHFR* or *OT 47 kDa*. The primer sequences were given in Table S1. Relative quantification analysis of qPCR data, based on comparative ΔΔCt method, was adopted to indirectly assess the degree of OT infection in each culture. *OT 47 kDA* and *MTHFR* were set as the target gene and the reference gene respectively. Finally, the relative number of *OT 47 kDa* per *MTHFR* in each culture at different time points was normalized to the mean *OT 47 kDa/MTHFR* ratio of infected cultures that did not expose to IFN-γ harvested at 6 hours p.i.. The normalized *OT 47 kDa/MTHFR* value was designated as “infection index”, which reflects the extent of intracellular harbour of OT in each culture condition.

### Statistical analysis

To reflect IDO1 activity, the proportion of L-Kyn to L-Trp was calculated for each sample. Differences in the levels of serum L-Trp, L-Kyn or L-Kyn/L-Trp ratio between any two groups of the subjects were evaluated using Mann-Whitney test. For continuous clinical and laboratory variables, their association with the levels of serum L-Trp, L-Kyn or L-Kyn/L-Trp ratio was assessed using Spearman correlation.

For experimental data, differences of L-Trp level, L-Kyn level, L-Kyn/L-Trp ratio, IDO1 expression, and infection indexes between any two culture conditions were evaluated using unpaired t-test with or without Welch's correction as appropriate.

## Results

### Clinical characteristics of the patients

Twenty patients with confirmed scrub typhus were enrolled into the study. Clinical and laboratory data of seventeen patients were available and were summarized in Table 1.

**Table 1:** Clinical and laboratory characteristics of the patients with scrub typhus.

Characteristics	Median (IQR)
Age	42 (27.5–62.0)
Sex (% of males)	82.4 (n = 14)
Fever day at presentation	7 (4–11)
Body temperature	38.0 (37.7–39.0)
Hospitalization (%)	76.5 (n = 13)
Eschar (%)	17.6 (n = 3)
Hepatosplenomegaly (%)	35.3 (n = 6)
Acute renal failure (%)	29.4 (n = 5)
Meningoencephalitis (%)	17.6 (n = 3)
Lymphadenopathy (%)	11.8 (n = 2)
Septic shock (%)	5.9 (n = 1)
DIC (%)	5.9 (n = 1)
WBC	10.36 (8.08–15.25)
Platelet	158 (10.55–20.55)
AST	61.5 (17–114.5)
ALT	31.5 (14–100)

IQR = interquantile range, DIC = disseminated intravascular coagulopathy, AST = aspartate aminotansferase, ALT = alanine aminotransferase.

### Functional activity of IDO1 in patients with scrub typhus

**Figure 1:**
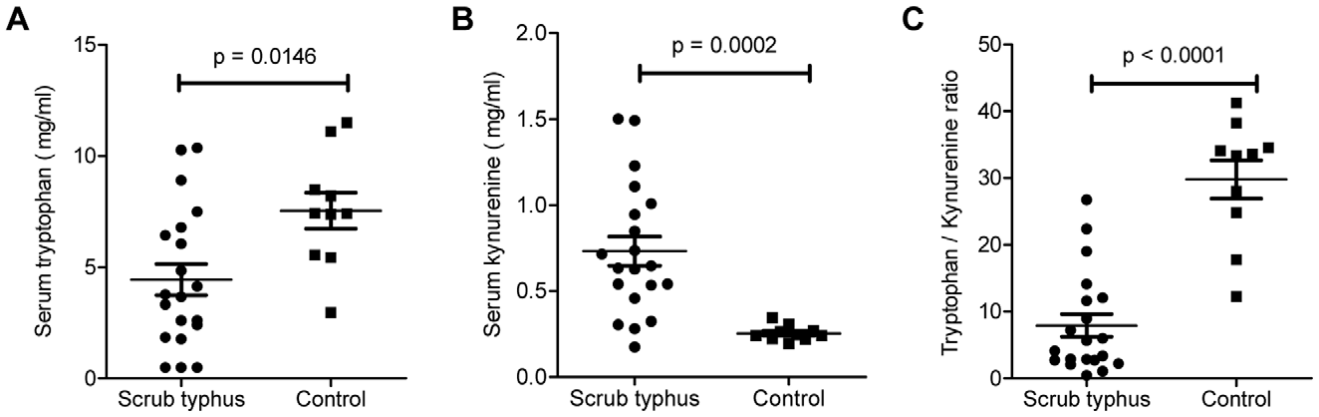
Assessment of IDO1 activity in patients' sera. Serum concentrations of L-Trp (A) and L-Kyn (B) in patients with scrub typhus (n = 20) were compared with those in healthy donors (n = 10). L-Kyn/L-Trp ratios (C) were calculated to reflect IDO1 activity. Data were derived from triplicate measurement. Median and interquantile range are presented.

To check whether functional activity of IDO1 enzyme actually increased in concordance to our earlier transcriptional study [10], serum levels of L-Trp and L-Kyn of patients with scrub typhus were determined (n = 20). As shown in Figure 1A and 1B, serum level of L-Trp was significantly lower in scrub typhus infected patients as compared to healthy individuals (*P* = 0.0146), whereas a reverse trend was observed for serum L- Kyn level (*P* = 0.0002). To see how IDO1 activity differs between the two groups of subjects, the ratio of L- Kyn to L-Trp was evaluated. As shown in Figure 1C, the enzyme activity in patients with scrub typhus was about nine times higher than that of healthy controls (*P*<0.0001).

Correlation analysis between levels of serum L-Kyn, serum L-Trp, or IDO1 activity and characteristics of the patients revealed no association for most of the clinical and laboratory parameters except for serum AST, whose level significantly correlates with serum L-Kyn (ρ = 0.6412, *P* = 0.0074 ) (Figure S1 and Table S2). When the patients were classified by their clinical features, no significant difference in levels of serum L-Kyn, serum L-Trp, or IDO1 activity was observed between any two subgroups of the patients. (Table S3)

### Expression of IDO1 in cell models

**Figure 2:**
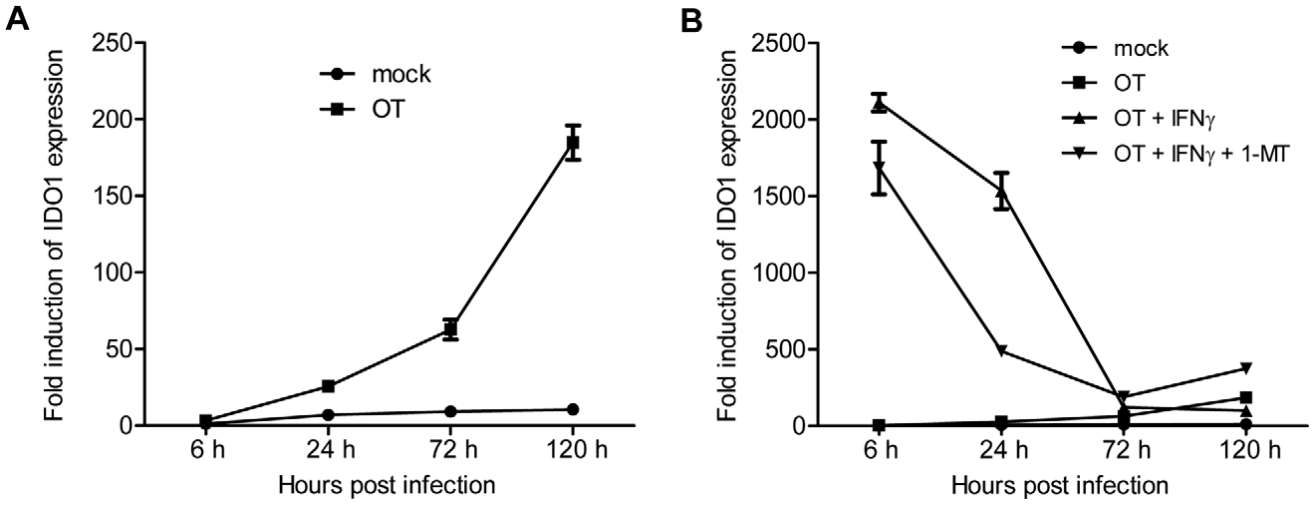
Kinetics of IDO1 expression in THP-1 cells. Before being infected with OT, THP-1 cells were treated with IFN-γ alone, IFN-γ combined with 1-MT, or neither. Mock-infected THP-1 cells were used as a control. Fold induction represents levels of IDO1 expression in each culture condition at indicated time points relative to that in mock-infected cells at 6 hours p.i.. For clearer illustration, data of OT-infected and mock-infected cultures are exclusively presented in Figure 2A, and those of all four culture conditions are shown in Figure 2B. Means ± SEM from triplicate cultures are shown.

To prove our hypothesis on the role of IDO1 in OT infection, we first evaluated temporal profiles of IDO1 gene transcription in an experimental model. As shown in Figure 2A, IDO1 expression was evidently induced in OT-infected THP-1 at 24 hours p.i. (*P*<0.0001) and increased up to 18 times greater than mock-infected cultures at 120 hour p.i. (*P* = 0.0001). IFN-γ treatment dramatically enhanced IDO1 transcription to even more than 2000 folds since 6 hours p.i. (*P*<0.0007), but the expression became comparable to that of unstimulated infected cultures at 72 and 120 hour p.i. (Figure 2B). The degree of IDO1 induction in cultures co-treated with IFN-γ and 1-MT was lower than those treated with IFN-γ alone at 6 and 24 hours p.i. (*P* = 0.077 and 0.001 respectively), yet overall kinetics of IDO1 expression in both conditions were quite similar (Figure 2B).

### Functional activity of IDO1 in THP1 cells

**Figure 3:**
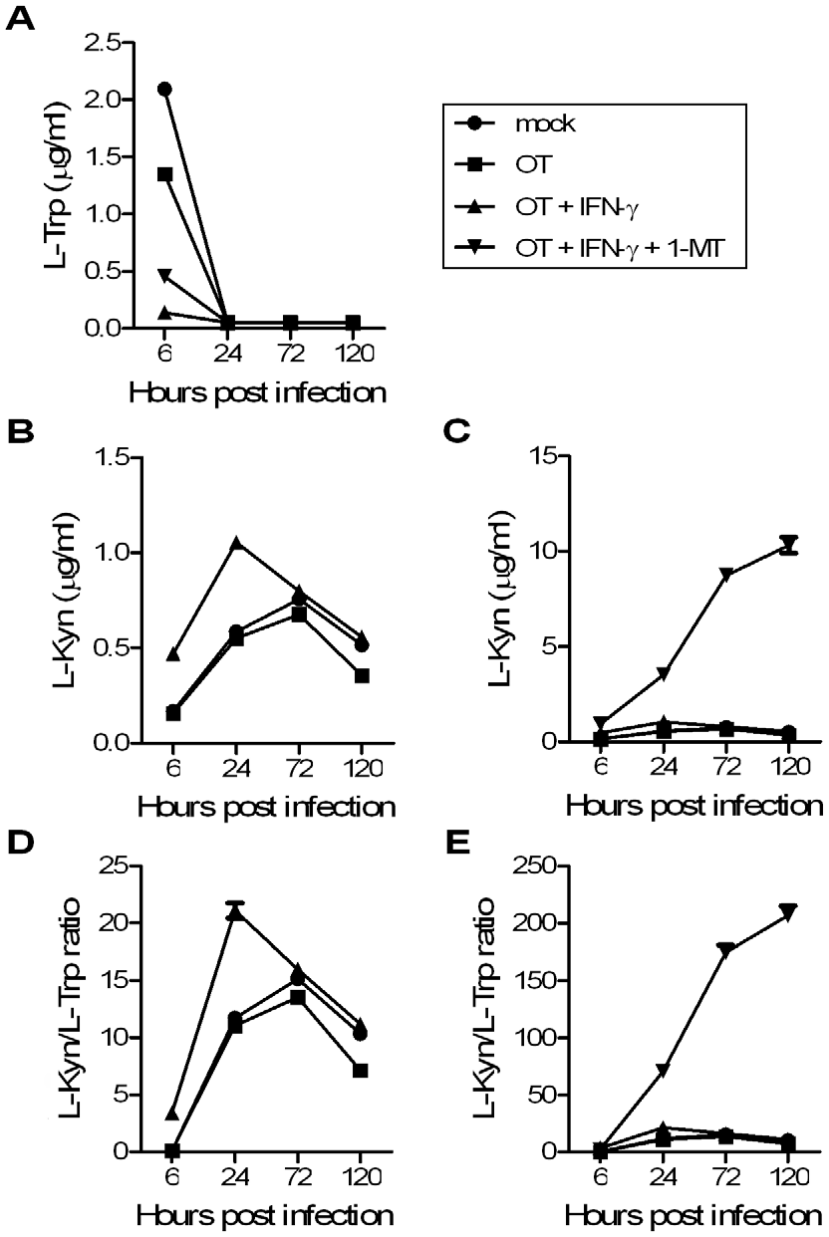
Assessment of IDO1 activity in OT-infected THP-1 cultures. Before being infected with OT, THP-1 cells were treated with IFN-γ alone, IFN-γ combined with 1-MT, or neither. Mock-infected THP-1 cells were used as a control. Levels of L-Trp (A) and L-Kyn (B, C) were measured in culture supernatant at 6, 24, 72, and 120 hours p.i.. IDO1 activity was reflected by the ratio of L-Kyn to L-Trp (D, E) at each indicated time point. Figure 3B and 3D represent the same dataset as Figure 3C and 3E respectively, but those treated with IFN-γ combined with 1-MT were excluded for clearer illustration. Means ± SEM from triplicate cultures are shown. Note: L-Trp concentration fell below a detectable level at 24, 48 and 72 hours p.i.; therefore, the lower limit of detection of L-Trp, 0.05 ng/ml, was used in the calculation of L-Kyn/L-Trp ratio for these time points.

To assess functional activity of IDO1 in a cell model, levels of L-Trp and L-kynurenine in culture supernatant of THP1 cells was determined at corresponding time points. As shown in Figure 3A, the level of L-Trp in OT-infected cultures was relatively lower than that of mock infection at 6 hours p.i. (*P* = 0.001) yet still significantly greater than that of IFN-γ treated ones (*P* = 0.0011). Co-treatment of IFN-γ and 1-MT retarded the rate of L-Trp consumption as compared with IFN-γ treatment alone at the same time point (*P* = 0.0334). However, the amino acid levels in culture supernatant fell below the limit of detection in all conditions by 24 hours p.i. (Figure 3A).

Despite the different rate of L-Trp consumption, the dynamic of L-Kyn level in OT-infected cultures was similar to that in mock-infected counterparts (Figure 3B and 3C). IFN-γ treatment significantly doubled L-Kyn level at the first 6 and 24 hours p.i. (*P* = 0.0027 and 0.0009 respectively), but the level became comparable to that of mock infection at later stages (Figure 3B). Unexpectedly, a continuous rise of L-Kyn level was observed in 1-MT treated cultures (Figure 3C). Since the difference of L-Trp levels among the four culture conditions was relatively unremarkable in comparison with that of L-Kyn levels, the profiles of L-Kyn/L-Trp ratio resembled that of L-Kyn as shown in Figure 3D and 3E.

### OT growth in cell cultures

**Figure 4:**
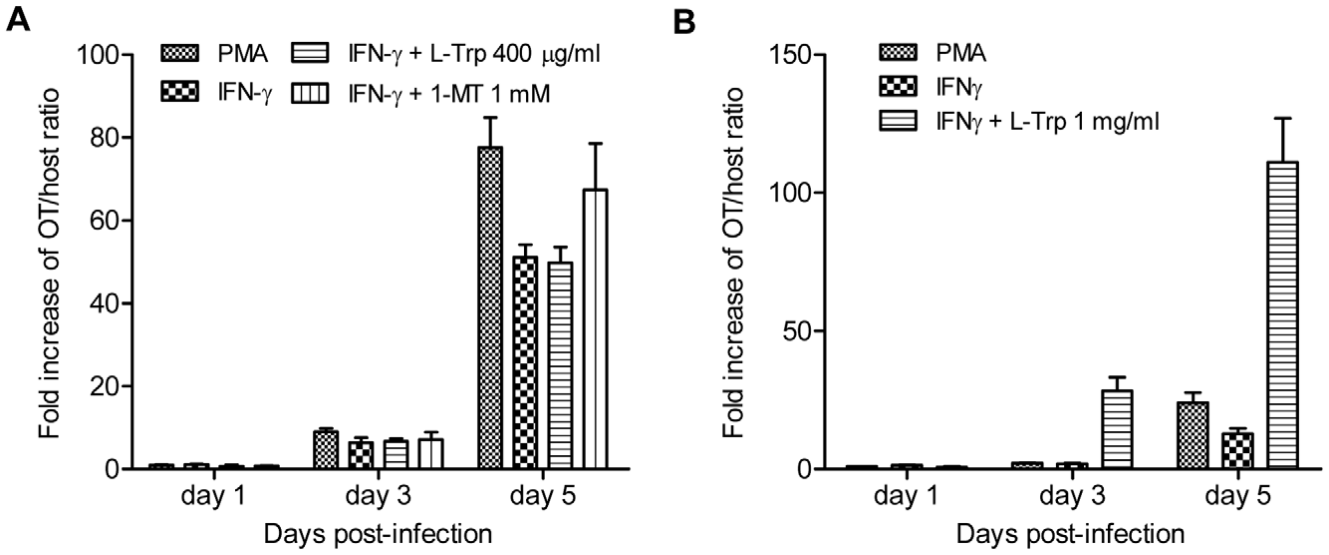
Assessment of OT growth in THP-1 cultures. Before being infected with OT, THP-1 cells were treated with IFN-γ alone, IFN-γ combined with 1-MT, or IFN-γ supplemented with L-Trp at 400 µg/ml (A) or 1 mg/ml (B). The infection index represents the degree of OT growth in each culture condition at indicated time points relative to that in mock-infected cells at 1 day p.i.. Means ± SEM from triplicate cultures are shown.

To investigate the effect of IDO1 activation on OT growth; an infection index, which is a relative copy number of OT 47 kDa gene per a human gene MTHFR, was determined in OT-infected cultures with or without IFN-γ-induced IDO1 activation. As shown in Figure 4A, the infection index was significantly lowered by IFN-γ treatment at 5 days p.i. (*P* = 0.0273). When IDO1 activity in IFN-γ treated cultures was inhibited by 1-MT, the infection index was partially restored. Replenishment of L-Trp in the culture media at a concentration of 400 µg/ml could not reverse the suppressive effect of IFN-γ-induced IDO1 induction on OT growth (Figure 4A). However, supplementation of the amino acid at 1 mg/µl markedly increased the infection index of IFN-γ-treated cultures to even higher than that the untreated counterparts as early as 3 days p.i. (*P* = 0.0326) (Figure 4B).

## Discussion

Activation of IDO1 is a host defensive mechanism downstream to IFN-γ that has been proven to limit the growth of various infectious pathogens in both immune and non-immune cells in vitro [12], [13], [17], [18], [19], [20]. It also appears to be induced in human subjects infected with some particular pathogens, such as *Leishmania guyanensis* [40], HIV-1 [37] and dengue virus [22]. According to the fact that OT lacks tryptophan-synthesizing enzyme [41], IDO1-mediated deprivation of tryptophan is a potential protective mechanism to limit the activity of this particular organism.

From our earlier analysis of genome-wide expression, we have observed an up-regulation of *IDO1* in peripheral blood leukocytes of patients with acute scrub typhus [10]. Thus, activation of IDO1 was first confirmed at functional level in patients with acute scrub typhus in the present study. Considering that monocytes appear to be the main source of active IDO1 in human peripheral blood [42], [43], THP-1 cells were used as an experimental model of infection in further testing of our hypothesis. Indeed, up-regulation of IDO-1 was previously demonstrated in OT-infected monocyte-derived macrophages [10]. Besides, we believe that macrophages could be a secondary target of infection to harbour the intracellular organisms to remote organs; which was supported by the detection of OT in circulating monocyte-like blood cells of patients with acute scrub typhus [48] and in macrophages located in the liver, the spleen, and the lymph nodes of fatal cases of scrub typhus [49], [50].

When THP-1 macrophages were infected with OT, transcription of IDO1 was directly induced by OT infection early after infection. However, a decreased level of L-Trp without a concomitant elevation of L-Kyn in OT-infected cultures as compared with mock infection may imply that higher rate of tryptophan consumption was contributed by increased utilization of the amino acid by the organism for its intracellular activity rather than enhancement of tryptophan catabolism by IDO1 enzyme. We postulated that the increase of IDO1 activity in vivo was a secondary event following the release of IFN-γ rather than a direct induction by the infection itself. For this reason, THP-1 was treated with IFN-γ prior to the in vitro infection to imitate natural infection in human body, in which other IFN-γ-producing cells such as NK cell, γδT cell, Th-1 cells and cytotoxic T cells, are also present. Such postulate seems sensible considering that the rise of IFN-γ during acute scrub typhus has been consistently observed by a number of earlier studies [7], [8], [9], [10].

After pre-treatment with exogenous IFN-γ, a marked increase in transcription of IDO1 along with its functional activity was detected as early as 6 hours p.i.. Concurrently, the intracellular number of OT was significantly depressed by IFN-γ treatment at 5 days p.i.. Even though IFN-γ is known to cause multiple changes in macrophage biology that could influence the outcome of an intracellular infection, our data suggest that IFN-γ-mediated IDO1 activation is responsible for growth restriction of OT to some extent since the number of OT per host cell was partially restored by a competitive inhibitor of the enzyme. However, it must be noted that induction of IDO1 only limited the rate of OT proliferation, but neither froze the growth nor reduced the number of the intracellular organism. For this reason, development of adaptive immune mechanisms seems to be required for eradication of the infection.

From earlier studies, anti-microbial activity of IDO1 has been explained by two non-mutually exclusive mechanisms: deprivation of tryptophan and formation of its downstream metabolites, collectively known as kynurenines. The first mechanism is based on the fact that tryptophan is the rarest essential amino acid in a human cell; therefore, IDO1-mediated tryptophan degradation, which limits the availability of the amino acid to be exploited by the organism, could lead to restraint of reproduction of tryptophan-sensitive microorganisms. In this study, we demonstrated that L-Trp supplementation at 400 µg/ml caused no change in the infection index of IFN-γ treated culture, whereas replenishment of the amino acid up to 1 mg/ml did not just restore but accelerated the growth of OT since the third day post infection. For this reason, it is not surprising why an earlier study that supplemented the culture media with 100 µg/ml of trytophan failed to rescue the growth of OT in IFN-γ-treated murine embryonic cell lines and refuted the role of tryptophan deprivation in control of OT infection [6]. Indeed, the number of the organisms in the culture with no IFN-γ treatment in the same experiment was demonstrated to be significantly increased by such small dose of tryptophan supplementation [6], which supported the dependence of OT proliferation on the availability of L-Trp similar to ours. Altogether, these data implies that IDO1-mediated tryptophan deprivation can indeed restrict OT growth; but restoration or enhancement of its reproduction requires adequate replenishment of tryptophan that allows an excess of the amino acid to be available in IDO1-active environment long enough for exploitation by this particularly slow-growing organism. However, it should be aware that comparing the findings derived from experiments in murine and human cell models might not be straightforward since defensive mechanisms to an infection may vary from species to species. For example, it was found that IDO1, but not iNOS, is essential in control of various infectious organisms in human mesenchymal stromal cells, while a reversed scenario is revealed for a similar cell type in mice [51]. In much the same way, it is possible that IDO1 is critical in control of OT growth in human macrophages but not in murine embryonic cells.

For the second mechanism involving IDO1-mediated kynurenine formation, it has been proved that some tryptophan metabolites, particularly 3-hydroxy-DL-kynurenine and alpha-picolinic acid but not L-Kyn, can exert anti-microbial activity against several extracellular bacteria in a dose-dependent manner [27]. In our experiments, the role of these metabolites in OT control was not directly investigated. However, supplementation of L-Trp into the culture, which was assumed to result in a greater amount of downstream metabolites produced in the system, did not increase the extent of OT growth restriction. Thus, we concluded that that formation of kynurenines was unlikely to play a major role in suppression of OT growth.

Despite the key findings that have already been discussed, we are also aware of some factors that could influence the interpretation of our experimental data. Firstly, the infection index, used as an indicator of OT growth in this study, was derived by a relative quantification method. It is, therefore, not only sensitive to a change in the number of OT but also to that of host cells at the time of the assessment. In the cultures supplemented with 1-mg L-Trp, we observed a reduction in the number of host cells by time, which might be partly responsible for a marked increase in the infection index of the condition compared with the others. Such cell loss could be a consequence of overwhelming expansion of OT in either tryptophan-rich environment or excessive accumulation of toxic metabolites in the culture system, or both. Secondly, the use of L-Kyn to L-Trp ratio as an indicator of IDO1 activity in THP-1 cells was unfortunately limited by the drop of L-Trp below the detectable level since the first day post infection. Moreover, some ambiguity in the data interpretation also resulted from the surprising rise of L-Kyn level in the culture supernatant of cells treated with 1-MT. Unlike other cultures whose L-Kyn level similarly reached its equilibrium at 72 hours p.i., the concentration of L-Kyn in 1-MT treated cultures continuously rose beyond 120 hour p.i.. This might reflect a higher rate of L-Kyn formation exceeding the capacity of downstream enzymes to catabolize the molecule into downstream metabolites even in the presence of the IDO1-specific inhibitor. However, it is unlikely that the other two tryptophan-degrading enzymes, IDO-2 and tryptophan 2,3-dioxygenase (TDO), were responsible for such phenomenon because the former appears to be expressed as an inactive form in human dendritic cells [52] and tumor cells [53], while the latter is exclusively expressed by hepatocytes [54]. Other possible explanations include an incidental blockage of a downstream kynurenine-degrading enzyme by 1-MT treatment, which results in accumulation of L-Kyn. However, further investigation is needed to determine the exact underlying mechanisms for such finding.

Apart from its anti-microbial activity, IDO1 also appears to be involved in suppression of immune responses as well as development of immunological tolerance. This concept originated from a key finding that demonstrated a role of IDO1-mediated tryptophan degradation in prevention of allogeneic fetal rejection [29]. It was later revealed that the enzyme exerts anti-proliferative effects on T cells, NK cells and tumor cells via degradation of tryptophan as well as production of its downstream metabolites, resembling its impact on microorganisms [30], [31], [32], [33]. Then, the issue has been actively concentrated in a variety of research areas, including infection, transplantation, autoimmunity, and cancer, with the hope to develop effective therapeutic strategies for these conditions. In certain dendritic cell subsets, IDO1 expression also appears to induce tolerogenic response to antigenic stimuli through other varieties of mechanisms, including induction of T cell anergy [34], apoptosis [35] and differentiation of regulatory T cell [36]. Based on our earlier study in patients with scrub typhus, such anti-proliferation of immune cells seemed unlikely to happen in vivo since we still observed marked leukocytosis, as well as up-regulation of genes in cell division process and leukocyte activation among the infected patients [10]. Nevertheless, further investigation is warranted to see whether IDO1 activation upon the infection also leads to tolerogenic responses or other modulation of the immune system.

Recently, a high level of serum L-Kyn/L-Trp ratio was reported to be associated with the development of severe complications, like septic shock and multiple organ failure, in patients with major trauma [55], sepsis [42], and bacteremia [56]. IDO1 activity during the course of the illness also appears to predict the severity and fatality for these patients [42], [55], [56]. According to its regulatory roles in the immune system as mentioned earlier, hyperactivity of IDO1 has been suspected to contribute to immune dysregulation that might underlie the development of such complications [55], [57]. Seeing that similar serious conditions are also common in severe cases of scrub typhus, activation of IDO1 might be involved in the development of such complications; its high functional activity could be a poor prognostic marker for this life-threatening disease as well. However, if we consider that activation of IDO1 would restrain the expansion of OT, an expected outcome would be in reverse. Considering that a positive correlation between the bacterial load in peripheral blood of scrub typhus patients and severity of the disease was recently reported [58]; it remains compelling to investigate a relationship between IDO1 activity and OT load, as well as, to reevaluate its association with clinical features in a larger set of patients with variable severity.

In conclusion, this is the first report for IDO1 activation in patients with scrub typhus, which has brought this multifaceted gene with promising therapeutic potential into focus in the field of scrub typhus research. We demonstrated here that IDO1-mediated tryptophan deprivation was a downstream mechanism of IFN-γ that helps restrain intracellular expansion of OT. However, further studies are deserved to investigate other potential effects of IDO1 activation on the outcome of OT infection in a more complex experimental model as well as in more patients with scrub typhus.

## Supporting Information

Figure S1 **A relationship between serum L-Kyn and AST in patients with scrub typhus (n = 16).** ρ = Spearman's rho.
(TIF)

Table S1 **Sequences of primer pairs for qPCR.**
(DOC)

Table S2 **Association between characteristics of the patients and levels of serum L-Trp, serum L-Kyn, and L-Kyn/L-Trp ratio.**
(DOC)

Table S3 **Levels of serum L-Trp, serum L-Kyn, and L-Kyn/L-Trp ratio in patients with scrub typhus classified by clinical characteritics.**
(DOC)
