# Supplementary figures and images for: Activation of Indoleamine 2,3-Dioxygenase in Patients with Scrub Typhus and Its Role in Growth Restriction of Orientia tsutsugamushi
Source: PLoS Negl Trop Dis. 2012 Jul 31;6(7):e1731. doi: 10.1371/journal.pntd.0001731 (PMC3409113; doi:10.1371/journal.pntd.0001731)

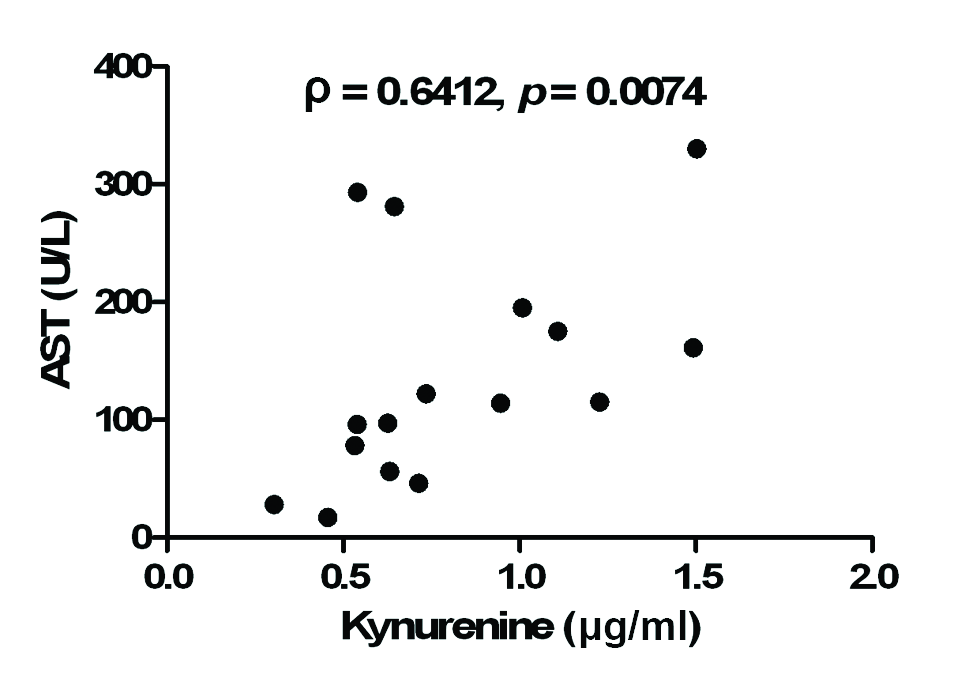

Supplement: Figure S1 — A relationship between serum L-Kyn and AST in patients with scrub typhus (n = 16). ρ = Spearman's rho. (TIF) [file pntd.0001731.s001.tif]
