# Supplementary material for: Activation of Indoleamine 2,3-Dioxygenase in Patients with Scrub Typhus and Its Role in Growth Restriction of Orientia tsutsugamushi
Source: PLoS Negl Trop Dis. 2012 Jul 31;6(7):e1731. doi: 10.1371/journal.pntd.0001731 (PMC3409113; doi:10.1371/journal.pntd.0001731)
Supplement: Table S1 — Sequences of primer pairs for qPCR. (DOC) [file pntd.0001731.s002.doc]

**Table S1.** Sequences of primer pairs for qPCR.

| Genes | Forward primers | Reverse primers | Product size (bp) |
| --- | --- | --- | --- |
| *TBP* | CAGGAGCCAAGAGTGAAGAA | CCTTATAGGAAACTTCACATCACAG | 143 |
| *IDO1* | CAAATCCACGATCATGTGAACC | AGAACCCTTCATACACCAGAC | 112 |
| *MTHFR* | AAGGAGAAGGTGTCTGCGGGCGC | AAGATCCCGGGGACGATGGGG | 128 |
| *^§^OT47kDa* | ATCTTACTCAGGCATAAGTT | TAACATACCACGACGAATTT | 97 |

^§^ The primer pair specific to 47 kDa-protein-encoding gene of *O. tsutsugamushi* was designed based on DNA sequences of Kato strain (accession no. L11697).
