# Supplementary material for: Activation of Indoleamine 2,3-Dioxygenase in Patients with Scrub Typhus and Its Role in Growth Restriction of Orientia tsutsugamushi
Source: PLoS Negl Trop Dis. 2012 Jul 31;6(7):e1731. doi: 10.1371/journal.pntd.0001731 (PMC3409113; doi:10.1371/journal.pntd.0001731)
Supplement: Table S2 — Association between characteristics of the patients and levels of serum L-Trp, serum L-Kyn, and L-Kyn/L-Trp ratio. (DOC) [file pntd.0001731.s003.doc]

**Table S2. A**ssociation between characteristics of the patients and levels of serum L-Trp, serum L-Kyn, and L-Kyn/L-Trp ratio.

|  | Serum L-Trp | | Serum L-Kyn | | Serum L-Kyn/L-Trp | |
| --- | --- | --- | --- | --- | --- | --- |
| Characteristics | ρ | p-value | ρ | p-value | ρ | p-value |
| Age | -0.11 | ns | 0.07 | ns | 0.25 | ns |
| Fever day at presentation | 0.09 | ns | 0.32 | ns | 0.07 | ns |
| Body temperature |  | ns | -0.10 | ns | 0.02 | ns |
| WBC | 0.03 | ns | -0.25 | ns | -0.03 | ns |
| Platelets | 0.00 | ns | -0.28 | ns | -0.17 | ns |
| AST* | 0.30 | ns | 0.64 | 0.0072 | 0.02 | ns |
| ALT* | 0.27 | ns | 0.39 | ns | -0.09 | ns |

***** One patient was excluded from the analysis due to unavailability of their serum transaminase level. ρ = Spearman’s rho; ns = no significance
