# Supplementary material for: Activation of Indoleamine 2,3-Dioxygenase in Patients with Scrub Typhus and Its Role in Growth Restriction of Orientia tsutsugamushi
Source: PLoS Negl Trop Dis. 2012 Jul 31;6(7):e1731. doi: 10.1371/journal.pntd.0001731 (PMC3409113; doi:10.1371/journal.pntd.0001731)
Supplement: Table S3 — Levels of serum L-Trp, serum L-Kyn, and L-Kyn/L-Trp ratio in patients with scrub typhus classified by clinical characteritics. (DOC) [file pntd.0001731.s004.doc]

**Table S3. L**evels of serum L-Trp, serum L-Kyn, and L-Kyn/L-Trp ratio in patients with scrub typhus classified by clinical characteritics.

|  | | Serum L-Trp | | Serum L-Kyn | | Serum L-Kyn/L-Trp | |
| --- | --- | --- | --- | --- | --- | --- | --- |
| Clinical characteristics | | Median (IQR) | p-value | Median (IQR) | p-value | Median (IQR) | p-value |
| Meningoencephalitis | Yes (n=3) | 6.43 (4.84-6.79) | ns | 0.53 (0.30-0.54) | ns | 0.08 (0.04-1.11) | ns |
|  | No (n=14) | 3.50 (2.27-7.85) |  | 0.72 (0.61-1.14) |  | 0.27 (0.12-0.38) |  |
| Hepatosplenomegaly | Yes (n=6) | 2.97 (2.27-6.15) | ns | 0.67 (0.61-0.67) | ns | 0.27 (0.15-0.37) | ns |
|  | No (n=11) | 4.84 (2.60-8.91) |  | 0.64 (0.46-1.11) |  | 0.14 (0.05-0.36) |  |
| Eschar | Yes (n=3) | 2.41 (0.05-3.67) | ns | 0.71 (0.64-1.11) | ns | 0.30 (0.18-22.16) | ns |
|  | No (n=14) | 5.45 (2.61-7.85) |  | 0.63 (0.51-1.07) |  | 0.16 (0.07-0.36) |  |
| Acute renal failure | Yes (n=5) | 4.84 (1.86-8.35) | ns | 0.54 (0.5-0.59) | ns | 0.11 (0.07-4.66) | ns |
|  | No (n=11) | 4.14 (2.60-7.50) |  | 0.95 (0.63-1.23) |  | 0.24 (0.07-0.36) |  |

IQR = interquantile range; ns = no significance
